# Biomarkers for diagnosis of childhood tuberculosis: A systematic review

**DOI:** 10.1371/journal.pone.0204029

**Published:** 2018-09-13

**Authors:** Toyin Omotayo Togun, Emily MacLean, Beate Kampmann, Madhukar Pai

**Affiliations:** 1 McGill International TB Centre, and Department of Epidemiology, Biostatistics and Occupational Health, McGill University, Montreal, QC, Canada; 2 Vaccines and Immunity Theme, Medical Research Council Unit The Gambia at the London School of Hygiene & Tropical Medicine, Atlantic Boulevard, Fajara, The Gambia; 3 Faculty of Infectious and Tropical Diseases, London School of Hygiene and Tropical Medicine, Keppel Street, London, United Kingdom; 4 Manipal McGill Centre for Infectious Diseases, Manipal University, Manipal, India; Indian Institute of Technology Delhi, INDIA

## Abstract

**Introduction:**

As studies of biomarkers of tuberculosis (TB) disease provide hope for a simple, point-of-care test, we aimed to synthesize evidence on biomarkers for diagnosis of TB in children and compare their accuracy to published target product profiles (TPP).

**Methods:**

We conducted a systematic review of biomarkers for diagnosis of pulmonary TB in exclusively paediatric populations, defined as age less than 15 years. PubMed, EMBASE and Web of Science were searched for relevant publications from January 1, 2000 to November 27, 2017. Studies using mixed adult and paediatric populations or reporting biomarkers for extrapulmonary TB were excluded. Study quality was assessed using the Quality Assessment of Diagnostic Accuracy Studies—2 (QUADAS-2) framework. No meta-analysis was done because the published childhood TB biomarkers studies were mostly early stage studies and highly heterogeneous.

**Results:**

The 29 studies included in this systematic review comprise 20 case-control studies, six cohort studies and three cross-sectional studies. These studies reported diverse and heterogeneous forms of biomarkers requiring different types of clinical specimen and laboratory assays. Majority of the studies (27/29 [93%]) either did not meet the criteria in at least one of the four domains of the QUADAS-2 reporting framework or the assessment was unclear. However, the diagnostic performance of biomarkers reported in 22 studies met one or both of the WHO-recommended minimal targets of 66% sensitivity and 98% specificity for a new diagnostic test for TB disease in children, and/or 90% sensitivity and 70% specificity for a triage test.

**Conclusion:**

We found that majority of the biomarkers for diagnosis of TB in children are promising but will need further refining and optimization to improve their performances. As new data are emerging, stronger emphasis should be placed on improving the design, quality and general reporting of future studies investigating TB biomarkers in children.

## Introduction

Childhood tuberculosis (TB) is estimated to constitute approximately 5% of the TB caseload in low TB burden countries compared to an estimated 20–40% in high-burden countries [1, 2]. However, notification of TB in children and subsequently deriving an accurate estimate of the disease burden remain notoriously inaccurate. This is primarily because of the greater challenge in confirming the diagnosis of paediatric TB due to the paucibacillary nature of TB disease in children and difficulty in obtaining good quality respiratory specimen [3, 4]. In support of this assertion, it is estimated that more than two-third of all childhood TB cases are either unreported or undiagnosed, while 96% of the 239,000 children who died from TB in 2015 were not on treatment [5, 6].

The sensitivity of sputum smear microscopy in childhood TB is less than 15%, even with optimized methods such as centrifugation of samples and use of fluorescent microscopy [7]. While culture of *Mycobacterium tuberculosis (M*.*tb)* in biological samples is more sensitive than smear microscopy, bacteriological confirmation of paediatric TB by both mycobacterial growth indicator tube (MGIT) liquid culture and Löwenstein-Jensen (LJ) solid media seldom exceeds 40%, including when using gastric aspirates and induced sputum [8, 9].

Although in adult studies the sensitivity and specificity of Xpert MTB/RIF (Cepheid, USA) is comparable to that of liquid culture [10–12], data from paediatric studies suggest that the sensitivity of Xpert is lower in children, and substantially lower among ambulant paediatric populations compared to paediatric inpatients [13–19]. In 2017, the World Health Organization (WHO) endorsed the use of Xpert MTB/RIF Ultra cartridge (Ultra), based on the findings from a large multi-centre non-inferiority diagnostic accuracy study in adults with signs and symptoms of pulmonary TB [20]. The study reported that Ultra had 5% higher sensitivity relative to Xpert MTB/RIF (95% CI: +2.7, +7.8) but 3.2% lower specificity (95% CI: -2.1, -4.7), with sensitivity gains highest among smear-negative, culture-positive patients and in HIV-infected patients [21]. Preliminary data on the accuracy of Ultra testing of sputum for diagnosis of pulmonary TB in hospitalized children reported that Ultra detected 75.3% of cases positive by culture on the same sample, while the performance of Ultra is comparable to that of Xpert amongst children with a positive Xpert, Ultra or TB culture [22].

Diagnosis of childhood TB remains challenging with the current routine clinical and laboratory diagnostic tools. Thus, the need for a new, preferably non-sputum based point-of-care (POC) diagnostic tool that could give a rapid and accurate diagnosis of TB disease in children is widely acknowledged. Although tests based on host immune response hold promise in this regard, no immune-diagnostic has been developed into a POC test than can distinguish between latent TB infection (LTBI) and TB disease, and more importantly between TB disease and other respiratory infections. Both the tuberculin skin test (TST) and interferon (IFN)-γ release assays (IGRA) fail to differentiate *M*.*tb* infection from TB disease [23–25].

Research into TB biomarkers has gained prominence due to the lack of suitable tests based on detection of the pathogen [26], and their potential for translation into a non-sputum based POC test [27]. The majority of studies investigating novel TB biomarkers utilized adult populations, while TB diagnostic research studies are traditionally conducted in adults with the findings usually extrapolated to children. It is unlikely that adult findings can be accurately extrapolated to paediatric populations given the considerable differences in clinical presentation, pathology and underlying immune responses to *M*.*tb* between adults and children [28]. However, studies of TB biomarkers in children are now emerging, and there is growing advocacy to include children as early as possible in research for new diagnostics with greater attention to addressing particular diagnostic challenges for children [29].

Therefore, the aim of this systematic review was to evaluate emerging biomarkers for diagnosis of TB in children aged less than 15 years, and to compare their diagnostic accuracy to the WHO-endorsed target product profiles (TPP) recommended for potential new diagnostics for TB in children [27].

## Materials and methods

### Search strategy and selection criteria

We conducted a systematic review of biomarkers and multi-marker biosignatures for diagnosis of active tuberculosis in exclusively paediatric study subjects, defined as age less than 15 years, in studies published between January 1, 2000 and November 27, 2017. A copy of the protocol for this systematic review is included in the Supporting Information (S1 File). PubMed, EMBASE, and Web of Science were searched for relevant publications. In the case of PubMed, searches including medical subject headings (MeSH), “text words” (tw) and titles (ti) were used. In PubMed, the ‘English’ filter was not used. For EMBASE and Web of Science, ‘English’ and ‘Human’ filters were used. For each database, the search term was transposed as appropriate. The PubMed search term used was as follows:

((((tuberculosis[ti] OR TB[ti]) AND (child*[tw] OR pediat*[tw])) ((("biological markers"[mesh] OR biological marker*[tw] OR biomarker*[tw] OR biosignature*[tw]) NOT (tumour*[tw] OR tumor*[tw] OR "tumor markers, biological"[mesh])) OR (miRNA[tw] OR microRNA[tw] OR proteom*[tw] OR transcriptom*[tw] OR immunoassay*[tw] OR immunoassay[mesh] OR LAM[tw] OR lipoarabinomannan*[tw] OR ("immunologic tests"[mesh] AND diagnos*[tw]) OR ((mycolic acid[tw] OR glycolipid*[tw]) AND (diagnos*[tw] OR detect*[tw])) OR (cytokine*[tw] AND diagnos*[tw]) )) NOT (animals[mesh] NOT humans[mesh])))

We structured the preparation and reporting of our systematic review according to the Preferred Reporting Items for Systematic Reviews and Meta-Analysis (PRISMA) guidelines [30]. Only studies of humans or that used human biological samples were eligible for inclusion. Biomarkers and multi-marker biosignatures, of immunological and microbiological origin, were included. Studies using adult or mixed adult and paediatric populations were excluded. Studies reporting biomarkers for extra-pulmonary TB (EPTB) detection were excluded. Index tests that required imaging techniques or detection from bacterial culture were excluded. As systematic reviews for interferon-gamma release assays (IGRA) and mycobacterial DNA (e.g. GeneXpert MTB/RIF, TB-LAMP) already exist [18, 31–33], these biomarkers were not included in our study. Studies published in English and French were eligible for inclusion. Publications were screened by title and abstract by two reviewers (TT, EM) before full-text screening. TT and EM conferred to determine appropriateness of all selected articles.

### Data extraction

The form utilized for data extraction was piloted for a separate systematic review (MacLean E. et al., unpublished) and further refined for this systematic review. For all eligible articles, double data extraction was performed by TT and EM using the structured Google form. A list of the fields for data extraction and the structured Google form used are included in the Supporting Information (S2 File).

### Assessment of study quality

Two reviewers (TT, EM) assessed the quality of the studies using specific sets of criteria within four domains of the Quality Assessment of Diagnostic Accuracy Studies– 2 (QUADAS-2) framework [34]. As per QUADAS-2 guidelines, the selected questions were those deemed most relevant for identifying biases for studies included in the review. Each criterion was classified as either “Yes”, “No”, or “Unclear” when applied to the information that is available in the publications, as described in Table 1.

**Table 1:** QUADAS-2 framework for quality assessment.

Domain 1: Patient selection [could their selection have introduced bias?] **Signaling question 1**: was a consecutive or random sample of patients enrolled? Yes: publication explicitly states sampling strategy is consecutive or random or describes it with sufficient clarity such that this conclusion is clearly evident. No: Convenience sampling; purposive sampling. Unclear: Inadequate description to conclusively know sampling was consecutive or random. Applicability: are there concerns that the included patients and setting do not match the review question?
Domain 2: Index test [could the conduct or interpretation of the index test have introduced bias?] **Signaling Question 2**: were the index test results interpreted without knowledge of the results of the reference standard? (i.e. blinding) Yes: Conduct/interpretation of the index test was blinded. No: Unblinded conduct/interpretation. Unclear: Inadequate description of blinding. Applicability: are there concerns that the index test, its conduct, or interpretation differ from the review question?
Domain 3: Reference standard [could the reference standard, its conduct, or its interpretation have introduced bias?] **Signaling Question 3**: is the reference standard likely to correctly classify the target condition? Yes: Culture-based reference standard (could be composite standard) OR citation of valid diagnostic algorithm, e.g. American Thoracic Society No: Reference standard did not include culture Unclear: Inadequate description of reference standard to understand procedures Applicability: are there concerns that the target condition as defined by the reference standard does not match the question?
Domain 4: Flow and timing [could the patient flow have introduced bias?) **Signaling Question 4**: were all patients included in the analysis? Yes: All patients, after exclusions, were given the index test and reference tests No: Unaccounted for patients, after exclusions, remain Unclear: Inadequate description of patient flow through study

Note: Within the QUADAS-2 framework, there are four main domains in which bias can arise. We have chosen to assess each domain using one signaling question. The notes about applicability are considerations to make when considering overall quality of study and writing of manuscript.

### Diagnostic accuracy

Target product profiles for new TB diagnostic tests in adults and children have been published [27]. We reviewed the diagnostic performances of the biomarkers, where reported, to highlight biomarkers that met the WHO-recommended minimal targets of 66% sensitivity and 98% specificity for a new diagnostic test for TB disease in children, and/or 90% sensitivity and 70% specificity for a triage test.

We summarized the evidence by a review of the methodological characteristics of the published studies including immunological properties of the biomarkers, clinical samples and assays required, as well as assessment of study quality and reported diagnostic accuracy as described above. All data generated during this study are available from the corresponding author on request.

## Results

**Fig 1:**
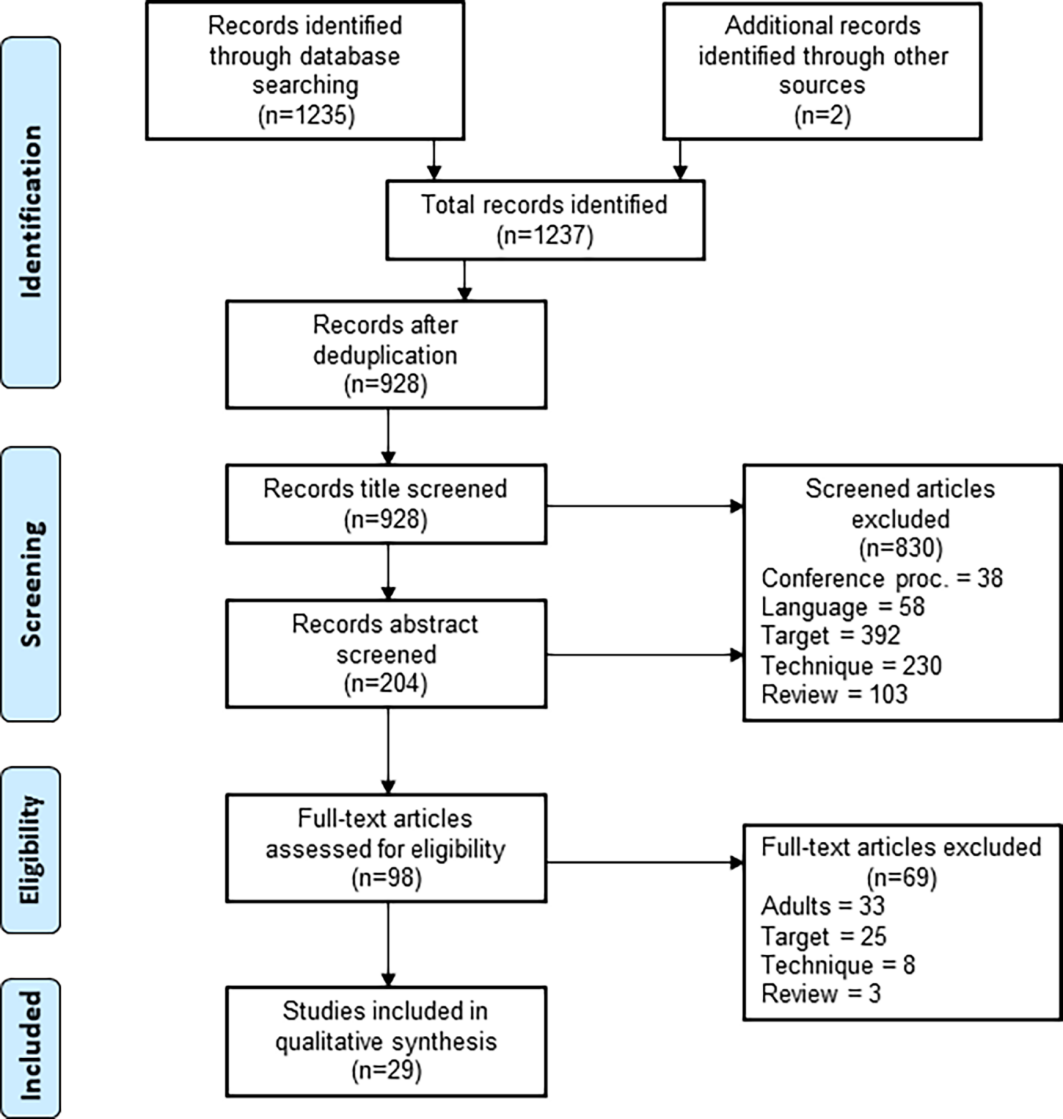
PRISMA flowchart. Flow diagram of inclusion and exclusion of studies. Reasons for exclusion are: conference proceedings and abstracts only; language (not English or French); technique (imaging-based, culture-based, commercial IGRA); reviews (narrative review, systematic review, meta-analysis); or target of paper (epidemiological, molecular biology, cost effectiveness, vaccine or drug study; biomarkers for detection of LTBI, prediction of disease progression or treatment monitoring). Studies that only included adult patients were also excluded.

A total of 1235 records were identified through database search for studies published between January 1, 2000 and November 27, 2017 and two additional records identified through reference lists. After removal of duplicates, 928 studies were screened using titles and abstracts; 98 full text articles were assessed for eligibility and 29 studies were eventually included in the systematic review (**Fig 1**).

### Characteristics of the studies

Table 2 summarizes the characteristics of the 29 studies in the systematic review, stratified by study design, including the statistical parameters used to assess diagnostic performance of the biomarkers when available [35–63]. Most of the studies (20/29 [69%]) were published between 2010 and 2017. Six of the published studies were cohort studies, while the others used either a cross-sectional (n = 3) or case-control (n = 20) study design. Thirteen of the studies were conducted in Asia (India = 7; China = 4; Bangladesh = 2), six in Europe, five in sub-Sahara Africa, three in the Americas, and one Australia. The 29 studies reported diverse and heterogeneous forms of biomarkers, which require different types of clinical specimen and utilized diverse techniques and laboratory assays for identifying biomarkers. The biomarkers include host-response markers comprising cytokine biomarkers (n = 7), cell surface biomarkers (n = 2), mRNA transcript signatures (n = 5), micro RNA signatures (n = 2), antibodies (n = 9), and metabolic signature (n = 1). Three studies investigated the utility of lipoarabinomannan (LAM), a mycobacterial cell wall antigen, for diagnosis of childhood TB.

**Table 2:** Study characteristics with measures of biomarker accuracy.

Author	Study title	Year Published	Journal	Sample/ patient location	Type of sample	Type of biomarker	Index test type	Sample size	Reference standard used	Negative population	Sensitivity (not %)	Specificity (not %)	AUC	Positive reference standard	Negative reference standard	Meets minimal TPP targets for triage and/or diagnostic test?
**COHORT STUDIES**																
Chiappini, E.[35]	Potential role of *M*. *tuberculosis* specific IFN-g and IL-2 ELISPOT assays in discriminating children with active or latent tuberculosis	2012	PLoS One	Spain	PBMCs	Cytokine (IL-2)	ELISPOT	75	Case definition according to American Academy Guidelines	LTBI	1.00 (95%CI:NR)	0.81 (95%CI:NR)	0.89 (95%CI: 0.79–1.00)	25	21	Meets both minimal TPP targets for triage test and one of the minimal TPP targets for a diagnostic test
^¥^Anderson S.T.[36]	Diagnosis of Childhood Tuberculosis and Host RNA Expression in Africa	2014	NEJM	South Africa, Malawi, Kenya	Whole blood	51-gene mRNA signature	Microarray	503	Culture	ORD	0.83 (95%CI: 0.69–0.94)	0.84 (95%CI: 0.75–0.93)	0.89 (95%CI: 0.82–0.95)	35	55	Meets one of the minimal TPP targets for a diagnostic or triage test
Portevin, D.[37]	Assessment of the novel T-cell activation marker-tuberculosis assay for diagnosis of active tuberculosis in children: a prospective proof-of-concept study	2014	Lancet Infect Dis	Tanzania	PBMCs	Haematological marker *(M*. *tuberculosis-specific* CD4+ CD27^+^ T-cells)	ICS/Flow cytometry	130	Culture	ORD	0.83 (95%CI: 0.59–0.96)	0.97 (95%CI: 0.89–0.99)	NR	18	63	Meets one of the minimal TPP targets for a diagnostic or triage test
Nicol, M.[38]	Urine lipoarabinomannan testing for diagnosis of pulmonary tuberculosis in children: a prospective study	2014	Lancet Glob Health	South Africa	Urine	*M*. *tuberculosis* cell wall antigen (Lipoarabinomannan)	ELISA and Lateral Flow Assay	535	Culture	ORD	ELISA: 0.02 (95%CI: 0.00–0.08) LFA: 0.48 (95%CI: 0.38–0.59)	ELISA: 0.96 (95%CI: 0.93–0.97) LFA: 0.61 (95%CI: 0.56–0.65)	ELISA: NR LFA: 0.56 (95%CI: 0.50–0.62)	89	446	ELISA-LAM meets one of the minimal TPP targets for a triage test; LFA does not meet either
Kroidl, I.[39]	Performance of urine lipoarabinomannan assays for paediatric tuberculosis in Tanzania	2015	Eur Respir J	Tanzania	Urine	*M*. *tuberculosis* cell wall antigen (Lipoarabinomannan)	ELISA and Lateral Flow Assay	132	Culture	ORD and LTBI	ELISA: 0.44 (95%CI:NR) LFA: 0.28 (95%CI:NR)	ELISA: 0.97 (95%CI:NR) LFA: 0.97 (95%CI:NR)	NR	18	35	ELISA-LAM and LFA-LAM meet one of the minimal TPP targets for a triage test
Tebruegge, M.[40]	Mycobacteria-Specific cytokine responses detect tuberculosis infection and distinguish latent from active tuberculosis	2015	Am J Respir Crit Care Med	Australia	Plasma	Cytokines (TNF-a/IL-10 & TNF-a/IL1-ra)	Multiplex	140	Case definition according to American Thoracic Society/CDC	LTBI	NR	NR	NR	6	16	NA
**CROSS SECTIONAL STUDIES**																
Raqib R.[41]	Detection of Antibodies Secreted from Circulating *Mycobacterium tuberculosis*-Specific Plasma Cells in the Diagnosis of Pediatric Tuberculosis	2009	Clin Vaccine Immunol	Bangladesh	PBMCs	Anti-BCG IgG	ALS/ELISA	132	Clinical consensus diagnosis	ORD and healthy controls	0.91 (95%CI:NR)	0.87 (95%CI:NR)	NR	58	74	Meets both minimal TPP targets for a triage test and one of the minimal TPP targets for a diagnostic test
Jenum S.[42]	Approaching a diagnostic point-of-care test for paediatric tuberculosis through evaluation of immune biomarkers across the clinical disease spectrum	2016	Sci Rep	India	Whole blood	mRNA signature (BPI, TNFRSF1B, CD3E, CD14, TIMP2, FPR1, IL4, TGFBR2)	dcRT-MLPA transcriptional assay	127	Clinical case definitions for childhood tuberculosis (under Revised National Tuberculosis Control Programme)	Asymptomatic household siblings	NR	NR	0.88 (95%CI:NR)	88	39	NA
Iskandar A.[43]	The Diagnostic Value of Urine Lipoarabinomannan (LAM) Antigen in Childhood Tuberculosis	2017	J Clin Diagn Res	Indonesia	Urine	*M*. *tuberculosis* cell wall antigen (Lipoarabinomannan)	ELISA	61	Composite reference standard based on clinical or microbiological confirmation.	ORD	0.86 (95%CI:NR)	0.83 (95%CI:NR)	0.80 (95%CI: 0.64–0.96)	49	12	Meets one of the minimal TPP targets for a diagnostic or triage test
**CASE CONTROL STUDIES**																
Simonney N.[44]	Analysis of circulating immune complexes (CICs) in childhood tuberculosis: levels of specific antibodies to glycolipid antigens and relationship with serum antibodies	2000	IJTLD	France	Serum	IgG anti glycolipid antigens in plain serum and Circulating immune complexes (anti-DAT IgG; anti-PGLTb1 IgG; anti-LOS IgG)	ELISA	53	Case definitions not specified	Non-tuberculous diseases	0.83 (95%CI:NR)	0.89 (95%CI:NR)	NR	12	26	Meets one of the minimal TPP targets for a diagnostic or triage test
Imaz M.S.[45]	Evaluation of the diagnostic value of measuring IgG, IgM and IgA antibodies to the recombinant 16-kilodalton antigen of *Mycobacterium tuberculosis* in childhood tuberculosis	2001	IJTLD	Argentina	Serum	Anti rec-Ag16 IgG, IgM and IgA	ELISA	272	Culture	Non-mycobacterial diseases	IgG + IgA: 0.43 (95%CI:NR)	IgG + IgA: 0.97 (95%CI:NR)	NR	29	149	Meets one of the minimal TPP targets for a triage test
Bhatia A.S.[46]	Serodiagnosis of Childhood Tuberculosis by ELISA	2005	Indian J Pediatri	India	Serum	Anti excretory-secretory-(ES)-31 IgG	ELISA	230	Case definitions not specified	ORD	0.83 (95%CI:NR)	0.93 (95%CI:NR)	NR	30	60	Meets one of the minimal TPP targets for a diagnostic or triage test
Dayal R.[47]	Diagnostic Value of Elisa Serological tests in childhood tuberculosis	2006	J Trop Pediatr	India	Serum	Anti-PGLTb1 IgG; anti ESAT-6 IgG	ELISA	197	Composite reference standard based on clinical or microbiological confirmation	ORD	Anti-PGLTb1 IgG: 0.53 (95%CI:NR) Anti-ESAT6 IgG: 0.59 (95%CI:NR)	Anti-PGLTb1 IgG: 0.93 (95%CI:NR) Anti-ESAT6 IgG: 0.85 (95%CI:NR)	NR	Anti-PGLTb1: 32 Anti-ESAT6: 37	Anti-PGLTb1: 27 Anti-ESAT6: 22	Meets one of the minimal TPP targets for a triage test
Kumar G.[48]	Diagnostic potential of Ag85C in comparison to various secretory antigens for childhood Tuberculosis	2008	Scand J Immunol	India	Serum	Anti Ag85C IgG	ELISA	147	Composite reference standard based on clinical or microbiological confirmation	ORD and health controls	0.90 (95%CI:NR)	0.92 (95%CI:NR)	0.98 (95%CI:NR)	36	42	Meets both minimal TPP targets for a triage test and one of the minimal TPP targets for a diagnostic test
Dayal R.[49]	Serological Diagnosis of Tuberculosis	2008	Indian J Pediatr	India	Serum	Anti Ag85 complex IgG	ELISA	115	Composite reference standard based on clinical or microbiological confirmation	ORD	0.61 (95%CI:NR)	0.67 (95%CI:NR)	NR	64	32	Did not meet either
Senol G.[50]	Humoral Immune Response Against 38- and 16-kDa mycobacterial antigens in childhood tuberculosis	2009	Pediatr Pulmonol	Turkey	Serum	Anti 16kDa IgG + Anti 32kDa IgG	ELISA	72	Composite reference standard based on clinical or microbiological confirmation	ORD and health control	0.25 (95%CI:NR)	0.90 (95%CI:NR)	NR	17	40	Meets one of the minimal TPP targets for a triage test
Lighter-Fisher J.[51]	Cytokine responses to QuantiFERON peptides, PPD and recombinant ESAT-6 in children with tuberculosis	2010	IJTLD	USA	Plasma	Cytokine (IL-2, TGF-β1 in QFT stimulation assay)	Multiplex	92	Case definitions not specified	LTBI	NR	NR	NR	11	46	NA
Mueller H.[52]	Granulysin-Expressing CD4^+^ T-cells as Candidate Immune Marker for Tuberculosis during Childhood and Adolescence	2011	PLoS One	Germany	PBMCs	Haematological marker (*M*. *tuberculosis-specific* CD4+ CD45RO^+^ T-cells co-expressing granulysin)	ICS/Flow cytometry	59	Case definitions not specified	NTM and healthy controls	NR	NR	NR	7	18	NA
Thomas T.[53]	A new potential biomarker for childhood tuberculosis	2011	Thorax	Bangladesh	Plasma	Anti BCG IgG	ALS/ELISA	132	Composite reference standard based on clinical or microbiological confirmation	ORD and healthy controls	0.91 (95%CI:NR)	0.87 (95%CI:NR)	NR	58	74	Meets both minimal TPP targets for a triage test and one of the minimal TPP targets for a diagnostic test
Gourgouillon, N.[54]	TNF-α/IL-2 ratio discriminates latent from active tuberculosis in immunocompetent children: a pilot study	2012	Pediatr Res	France	Plasma	Cytokines (TNF-α/IL-2 ratio)	Multiplex	18	Case definitions not specified	LTBI	0.88 (95%CI:NR)	0.83 (95%CI:NR)	NR	8	6	Meets one of the minimal TPP targets for a diagnostic or triage test
Verhagen L.M.[55]	A predictive signature gene set for discriminating active from latent tuberculosis in Warao Amerindian children	2013	BMC Genomics	Venezuela	Whole blood	5-gene mRNA signature	Microarray	81	Composite reference standard based on clinical or microbiological confirmation	LTBI, healthy controls and non-TB pneumonia	0.78 (95%CI:NR)	0.96 (95%CI:NR)	0.94 (95%CI:NR)	9	72	Meets one of the minimal TPP targets for a diagnostic or triage test
Kumar N.P.[56]	Circulating Biomarkers of Pulmonary and Extrapulmnonary Tuberculosis in Children	2013	Clinical and Vaccine Immunology	India	Plasma	Cytokines (TGF-β; IL-21; IL-23	Multiplex	55	Culture	Health controls	NR	NR	NR	14	19	NA
Chegou N.N.[57]	Utility of Host Markers Detected in Quantiferon Supernatants for the Diagnosis of Tuberculosis in Children in High-Burden Setting	2013	PLoS One	South Africa	Plasma	Cytokines (IL-1ra, IL-1α, IP-10, sCD40L and TNF-α)	Multiplex	76	Culture	LTBI and uninfected controls	0.84 (95%CI:NR)	0.84 (95%CI:NR)	NR	19	47	Meets one of the minimal TPP targets for a diagnostic or triage test
Dhanasekaran S.[58]	Identification of biomarkers for Mycobacterium tuberculosis infection and disease in BCG-vaccinated young children in Southern India	2013	Genes and Immunity	India	Whole blood	mRNA signature (RAB33A, CXCL10, SEC14L, FOXP3 and TNFRSF1A)	RT-MLPA	210	TB paediatric diagnostic algorithm	Uninfected controls	NR	NR	0.92 (95%CI:NR)	13	107	NA
Armand M.[59]	Cytokine responses to Quantiferon peptides in pediatric tuberculosis: A pilot study	2014	J Infect	France	Plasma	Cytokines (IP-10; IL-2; IL-5; IL-13)	Multiplex	47	Case definition (NIH consensus, Graham et al 2012)	Healthy controls	IP-10: 0.95 (95%CI:NR) IL-2: 0.83 (95%CI:NR) IL-5: 0.90 (95%CI:NR) IL-13: 0.90 (95%CI:NR)	IP-10: 1.00 (95%CI:NR) IL-2: 1.00 (95%CI:NR) IL-5: 0.86 (95%CI:NR) IL-13: 1.00 (95%CI:NR)	IP-10: 0.96 (95%CI: 0.91–1.00) IL-2: 0.98 (95%CI: 0.93–1.01) IL-5: 0.91 (95%CI: 0.81–1.01) IL-13: 0.97 (95%CI: 0.93–1.00)	40	7	IP-10 and IL-13 meet both minimal TPP targets for diagnostic and triage tests; IL-2 meets both minimal TPP targets for diagnostic test; IL-5 meets both minimal TPP targets for a triage test
Wang J.X.[60]	Diagnostic values of microRNA-31 in peripheral blood mononuclear cells for pediatric pulmonary tuberculosis in Chinese patients	2015	Genet Mol Res	China	PBMCs	microRNA-31	RT-PCR	125	Clinical diagnostic criteria for paediatric tuberculosis, (Pan JH et al 2014)	Healthy controls	0.99 (95%CI:NR)	0.87 (95%CI:NR)	0.97 (95%CI: 0.93–0.99)	65	60	Meets both minimal TPP targets for a triage test and one of the minimal TPP targets for a diagnostic test
Li Q.[61]	Increased IL-9 mRNA expression as a biomarker to diagnose childhood tuberculosis in a high burden setting.	2015	J Infect	China	PBMCs	IL-9 mRNA	qPCR	64	Case definition not specified	Healthy controls	0.69 (95%CI:NR)	0.96 (95%CI:NR)	0.92 (95%CI: 0.83–1.00)	13	25	Meets one of the minimal TPP targets for a diagnostic or triage test
^¥^Sun L.[62]	Utility of Novel Plasma Metabolic Markers in the Diagnosis of Pediatric Tuberculosis: A Classification and Regression Tree Analysis Approach	2016	J Proteome Res	China	Plasma	Metabolic signature (L-valine, pyruvic acid and betaine)	^1^HNMR Spectroscopy	113	Case definition not specified	ORD and healthy controls	0.82 (95%CI: 0.56–0.95)	0.84 (95%CI: 0.66–0.94)	0.80 (95%CI: 0.65–0.94)	17	31	Meets one of the minimal TPP targets for a diagnostic or triage test
Zhou M.Y.[63]	Circulating microRNAs as biomarkers for the early diagnosis of childhood tuberculosis infection	2016	Mol Med Rep	China	PBMCs	microRNA signature (μRNA-1, μR-10a, μRNA-31, μRNA-125b, μRNA-146a, μRNA-150, μRNA-155, μRNA-29b)	Microarray	98	Case definition not specified	Healthy controls	0.96 (95%CI:NR)	1.00 (95%CI:NR)	0.99 (95%CI: 0.91–1.00)	25	21	Meets both minimal TPP targets for diagnostic and triage tests

AUC: Area under the receiver operating characteristics curve; PPV: positive predictive value; NPV: negative predictive value; Positive reference standard: number of TB cases assayed; Negative reference standard: number of reference standard negative controls; PBMCs: peripheral blood mononuclear cells; LTBI: latent TB infection; NR: not reported; NTM: Non-tuberculous mycobacteria; ORD: other respiratory diseases; ELISA: Enzyme-linked immunosorbent assay; ELISPOT; Enzyme linked immuno-spot assay; dcRT-MLPA: dual colour Reverse-Transcriptase Multiple Ligation-dependent Probe Amplification; mRNA: messenger RNA; ICS: intracellular cytokine staining; ALS: antibodies in lymphocyte supernatants; RT-PCR: real time polymerase chain reaction; qPCR: quantitative polymerase chain reaction; LAM: Lipoarabinomannan.

^¥^Reported biomarker performance determined in an external validation cohort

### Study quality

**Fig 2:**
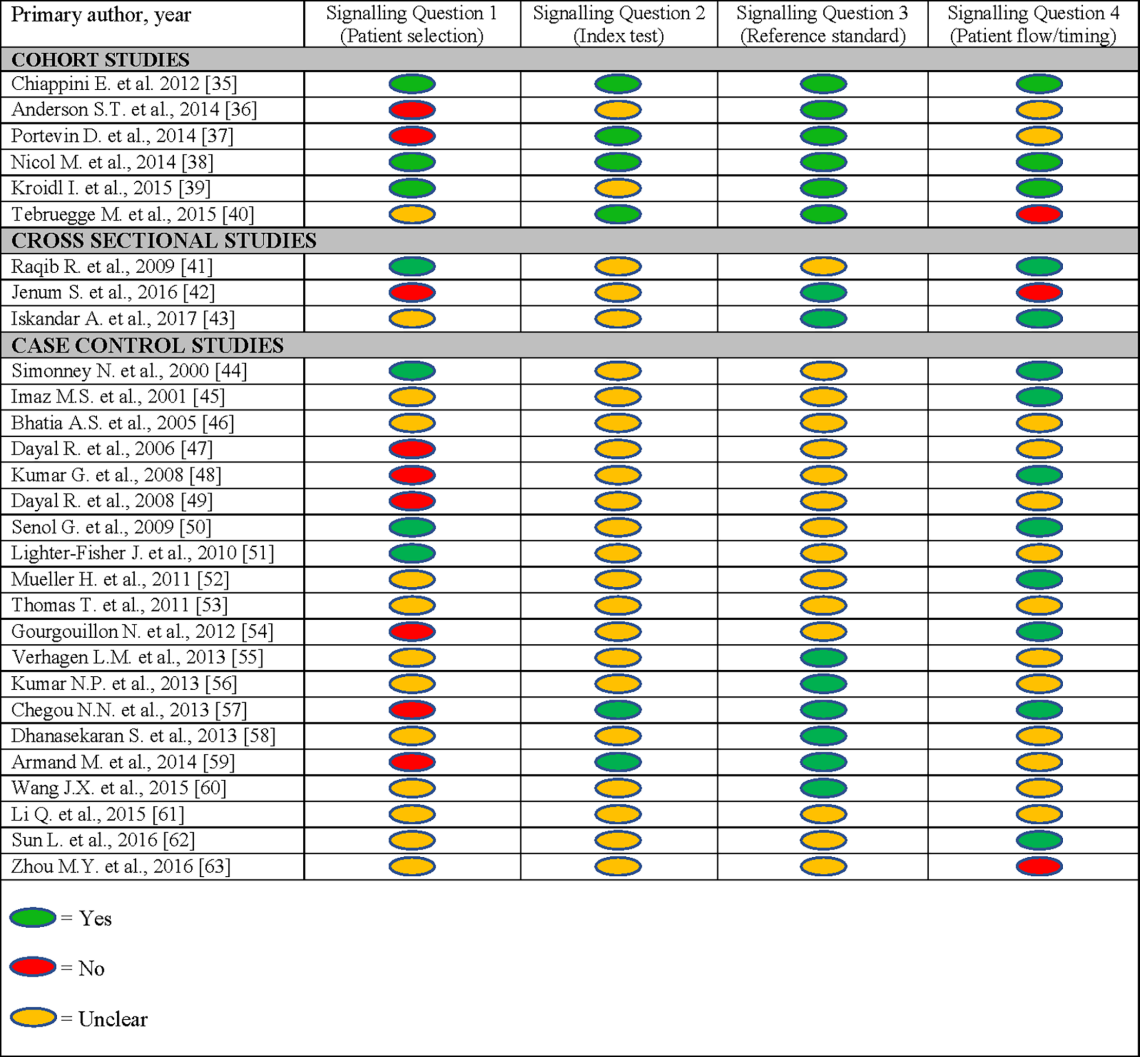
Quality assessment of childhood TB biomarker studies. Results from assessment of the quality of each study in the systematic review using QUADAS-2 framework.

Fig 2 shows the results from assessment of the quality of the studies in our systematic review using four questions from the QUADAS-2 framework.

**Patient selection** (*i*.*e*. *was a consecutive or random sample of patients enrolled*?). Out of the six cohort studies, three publications explicitly stated that consecutive samples of eligible subjects were recruited [35, 38, 39]. Two studies were deemed to have used purposive sampling by conducting biomarker analysis on a sub-selection of eligible subjects because of cost [37], or by excluding IGRA positive children with other respiratory diseases in their discovery cohort [36]. The sampling strategy was not clearly described in the study by Tebruegge et al [40]. Only one cross-sectional study and three out of 20 case-control studies reported that a consecutive sample of eligible study subjects were enrolled [41, 50–52]. The remaining studies used either convenience or purposive sampling strategy, or their sampling strategy was inadequately described.

**Index test** (*i*.*e*. *was the conduct and interpretation of the index test blinded*?). Four out of six cohort studies explicitly stated that the conduct and interpretation of the index test was blinded [35, 37, 38, 40]. None of the cross-sectional studies, and only two case-control studies reported blinding of the conduct and interpretation of the index test results [57, 59].

**Reference standard** (*i*.*e*. *is the reference standard likely to correctly classify the target condition*?). All six cohort studies reported either a culture-based reference standard or a composite reference standard with citation of a valid diagnostic algorithm. In contrast, two of the three cross-sectional studies [42, 43], and six of the 20 case-control studies reported such a reference standard [45, 56–60].

**Patient flow and timing** (*were all patients included in the analysis*?). All study subjects, after exclusions, were given the index and reference tests in three cohort studies [35, 38, 39], while some subjects could not be accounted for after exclusions in one study [40]. The description was inadequate to clearly ascertain patient flow and timing in the other two cohort studies [36, 37]. Two cross-sectional and eight case-control studies showed that all patients were given the index and reference tests after exclusions. One cross-sectional study and one case-control study had patients that could not be accounted for after exclusions, while the description of patient flow and timing was unclear in 11 case-control studies (Fig 2).

### Diagnostic performance of biomarkers

Table 2 shows the accuracy estimates from all the studies that reported such data. Cytokine biomarkers (IP-10, IL-2 and IL-13) in a case-control study by Armand *et al* demonstrated sensitivity ≥ 80% and specificity ≥ 98%, while circulating microRNAs in another case-control study by Zhou *et al* demonstrated sensitivity and specificity of 96% and 100% respectively [59, 63]. These biomarkers met both recommended minimal targets for a new diagnostic test.

Seven studies reported biomarkers that met both minimal TPP targets for a new triage test. These include an IL-2 ELISPOT assay using recombinant *M*.*tb* antigen (secreted L-alanine dehydrogenase [AlaDH]) that distinguished active TB and LTBI in a prospective study with a sensitivity of 100% and specificity of 81% [35]. Also, anti-BCG IgG secreted from *M*.*tb*-specific plasma cells in a cross-sectional study of a new serological method (antibodies in lymphocyte supernatants) that distinguished children with TB and other diseases demonstrated sensitivity and specificity of 91% and 87% respectively [41]. Anti-Ag85C IgG [48], anti-BCG IgG [53], microRNA-31 [60], circulating microRNAs [63], and cytokine biomarkers (IP-10, IL-5 and IL-13) [59] reported in five of the case-control studies also met both minimal targets for a triage test. However, 15 studies reported biomarkers that met just one of the minimal TPP targets for a diagnostic or triage test. These biomarkers had sensitivity greater than 66% but specificity less than the minimum of 98% set for a diagnostic test, or demonstrated sensitivity less than 90% but specificity that exceed the minimum of 70% for a new triage test [36–39, 43–47, 50, 54, 55, 57, 61, 62].

## Discussion

The investigation and development of new TB diagnostics that are suitable for children has been highlighted as a research priority for the End TB Strategy by the WHO [64]. We conducted a systematic review of host-response and pathogen-derived biomarkers for diagnosis of pulmonary TB disease in children, assessed quality of the included studies using the standardised QUADAS-2 framework, and compared the diagnostic performances of the candidate biomarkers to the published TPP recommended for new diagnostics for TB in children. In general, we found that the published childhood TB biomarkers studies were mostly early-stage studies and highly heterogeneous in terms of the specific type of biomarkers, clinical samples, test methods, and reference standards used for diagnosis of pulmonary TB disease in children. Therefore, we did not perform a meta-analysis.

An optimally designed diagnostic accuracy study is a prospective study with a blind comparison of the index and reference tests in consecutively recruited study subjects from a relevant clinical population [65, 66]. If a diagnostic or triage test is to be applied in multiple settings globally, then an optimal diagnostic accuracy study should also be multi-centre and/or performed in multiple diverse geographical locations and populations. The majority of the studies included in our systematic review used a case-control study design with the selection of children defined as having TB disease and comparison groups that include healthy uninfected or *M*.*tb* infected controls in most cases. Other studies enrolled children with suspected TB disease referred for investigations to ascertain their diagnosis, using either a cohort or cross-sectional study design.

In our assessment of study quality, we found that most case-control studies were at an unclear or high risk of bias. Included case-control studies typically either did not utilize a consecutive sampling strategy or the sampling strategy was unclear. Generally, it was unclear if reported biomarker results were interpreted without knowledge of the results of the reference standards. Most of the cohort and cross-sectional studies that recruited children with suspected TB disease were also found to be at risk of bias because they either did not meet the criteria or assessment was not clear in at least one QUADAS-2 domain.

The risk of overestimating diagnostic accuracy is much higher in studies that use a case-control study design compared to other designs [67, 68]. A meta-analysis that investigated the importance of 15 design features on estimates of diagnostic accuracy reported *a relative diagnostic odds ratio* (RDOR) of 4.9 especially in case control studies that included healthy controls [69]. This mean diagnostic accuracy studies, particularly with the inclusion of healthy controls, are likely to overestimate the diagnostic performance almost five times. The inclusion of healthy controls introduces a design deficiency with lower occurrence of false-positive results and thus increasing the specificity [70].

The lack of a sensitive and specific reference standard for TB disease in children and of standardized case definitions are known to constitute major challenges to the assessment of accuracy of new diagnostic tools for childhood TB and for comparison of findings between diagnostic studies [71, 72]. Therefore, we compared the reported diagnostic performances of the biomarkers in our systematic review to the minimal targets of diagnostic performance recommended in a WHO-endorsed TPP for new TB diagnostic tests in children [27]. For a new diagnostic test in children, a sensitivity of ≥ 66% for intrathoracic TB is considered optimal, as this can currently be achieved using appropriate samples with Xpert, while the specificity should be ≥ 98% specificity of a microbiological reference standard [18, 27]. The sensitivity of a triage test should ideally be as high as that of the confirmatory test, but if a triage test could be conducted at lower levels of care and is easier to do, then conceivably more children with a higher likelihood of TB disease will be identified even if its sensitivity is lower than that of confirmatory test [27]. As such, the minimal sensitivity and specificity for a new triage test were set at 90% and 70% respectively, in order to make such triage testing potentially cost-effective in an implementation strategy.

Two case-control studies by Armand *et al* and Zhou *et al* reported cytokine biomarkers and circulating microRNAs respectively, which met both minimal sensitivity and specificity targets for a diagnostic or triage test [59, 63]. Overall, majority of the studies in this review reported biomarkers that met one or both of the minimal sensitivity and specificity TPP targets for use either as a diagnostic or triage test in children, which makes them promising. Biosignature thresholds are often set to obtain an optimum accuracy using Receiver Operating Characteristic (ROC) analysis [73, 74]. It is possible to re-optimise such thresholds for the biomarkers that met just one of the minimal TPP targets, which could further increase the sensitivity or specificity of the biomarkers toward meeting both targets either for a diagnostic or triage test. However, these results should be interpreted cautiously while taking into consideration the assessment of the quality of the individual studies and the potential for overestimation of diagnostic performance. In particular, findings from the case-control studies were deemed to have a high risk of bias from assessment of their quality with very probable overestimation of the reported diagnostic performances as discussed earlier.

A number of the studies in this review investigated and reported the utility of antibodies for diagnosis of TB disease in children, including a novel serological assay called antibodies in lymphocyte supernatant [41, 53]. Although the WHO encourages research in serological tests, the organization has recommended against the use of the currently available commercial antibody-based tests for TB diagnosis [75].

Critically, the failure of almost all studies to clearly articulate the intended use case of their biomarker-based diagnostic test, and to benchmark a biomarker towards it, has been highlighted as one of the key issues that limit the impact and translation of biomarkers into new diagnostic tests [76]. None of the studies in our systematic review clearly stated the intended use of the reported biomarkers either as a diagnostic or triage test in children. Furthermore, it has been suggested that an “ideal” biomarker (or set of biomarkers) that could be developed into an accurate test for TB in children should fulfil the following characteristics: (i) measurable in small volumes of readily obtainable samples such as blood, urine, stool, saliva, etc.; (ii) identify *M*.*tb* with high sensitivity and specificity regardless of age, nutritional status or HIV status; (iii) distinguish children with active TB disease from latently infected children with other respiratory infections; and (iv) suitable for incorporation into a diagnostic platform that would provide rapid results at or near the point of care [77]. While the performance of majority of the biomarkers in this review is promising, most of the biomarkers will need further refining and optimization while taking into consideration these methodological characteristics of an “ideal” biomarker. As such, the biomarkers should be evaluated in stronger and better-designed prospective studies to limit risk of bias and to assess the feasibility of incorporating them into diagnostic platforms implementable in high TB burden settings.

Our systematic review has limitations. A formal assessment of publication bias was not performed; existing methods like funnel plots or regression tests are not helpful for diagnostic accuracy studies [78]. Additionally, it is always possible in a systematic review that relevant publications were not identified in the search. However, the search term was constructed with the assistance of a medical librarian, and the term was adapted from a previously used term that was extensively calibrated (MacLean E. et al, unpublished).

## Conclusions

The fact that most of the studies investigating TB biomarkers in children were published within the last seven years supports the assertion that such data are now emerging. However, the results from this systematic review suggest that stronger emphasis need to be placed on improving the design, quality, and general reporting of studies investigating childhood TB biomarkers. In particular, future research studies in this area should target their biomarker research toward the TPP for a new diagnostic and/or triage test intended for use in children. In addition, such studies should be multi-centre studies performed in diverse geographical locations and populations, such that the diagnostic or triage test can be applied in multiple settings globally. Another approach is to conduct side-by-side/parallel diagnostic accuracy studies using prospective cohorts to benchmark different triage or diagnostic tests performance against each other. These will enhance the reliability, comparability, and reproducibility of the results, as well as the potential to translate the findings to the clinic while promoting more collaborative research. We hope that this systematic review will contribute to provide targeted guidance for further scientific explorations toward the eventual development of the next-generation POC test for the rapid and accurate diagnosis of TB disease in children.

## Supporting information

S1 File Systematic review protocol. A copy of the protocol for this systematic review.
(DOCX)

S2 File Supplementary methods. List of the fields for data extraction and structured google form used for data extraction.
(PDF)

S1 Table PRISMA checklist. Checklist according to the PRISMA reporting guidelines.
(DOC)
