# Supplementary material for: Biomarkers for diagnosis of childhood tuberculosis: A systematic review
Source: PLoS One. 2018 Sep 13;13(9):e0204029. doi: 10.1371/journal.pone.0204029 (PMC6136789; doi:10.1371/journal.pone.0204029)
Supplement: S1 File — A copy of the protocol for this systematic review. (DOCX) [file pone.0204029.s001.docx]

**SYSTEMATIC REVIEW PROTOCOL**

**Title:** **Biomarkers for diagnosis of childhood tuberculosis: a systematic review**

Toyin Togun^1^*, Emily MacLean^1^, Beate Kampmann^2,3^, and Madhukar Pai^1,4^

Author affiliations:

1. McGill International TB Centre, and Department of Epidemiology, Biostatistics and Occupational Health, McGill University, Montreal, QC, Canada.
2. Vaccines and Immunity Theme, Medical Research Council Unit The Gambia at the London School of Hygiene & Tropical Medicine, Atlantic Boulevard, Fajara, The Gambia.
3. Faculty of Infectious and Tropical Diseases, London School of Hygiene and Tropical Medicine, Keppel Street, London, United Kingdom.
4. Manipal McGill Centre for Infectious Diseases, Manipal University, Manipal, India.

*Corresponding author and address:

**Dr. Toyin Togun, MD, MPH, PhD**

McGill University

Department of Epidemiology, Biostatistics and Occupational Health

1020 Pine Avenue West

Montreal, QC H3A 1A2, Canada

E-mail: [toyin.togun@mail.mcgill.ca](mailto:toyin.togun@mail.mcgill.ca)

**background and RATIONALE**: Childhood tuberculosis (TB) is estimated to constitute approximately 5% of the TB caseload in low TB burden countries compared with an estimated 20% - 40% of the TB case load in high-burden countries (1, 2). However, the diagnosis and reporting of TB in children and establishing an accurate estimate of disease burden remains a daunting task principally because of the greater challenge in diagnosis of TB in children (3, 4). The sensitivity of smear microscopy in childhood TB remains less than 15% even with advances in performance of smear microscopy, such as concentration of specimens by centrifugation and the use of the relatively newer fluorescent microscopy with auramine-phenol staining (5). While culture of *Mycobacterium tuberculosis (M.tb)* in biological samples including sputum is more sensitive than smear microscopy, bacteriological confirmation of TB in children by both mycobacterial growth indicator tube (MGIT) liquid culture and Löwenstein-Jensen (LJ) solid media seldom exceeds 30% even when using gastric aspirates and induced sputum (2, 6-8). Although the sensitivity and specificity of Xpert MTB/Rif is comparable to that of liquid culture in adult studies (9, 10), emerging data from paediatric studies suggests that the sensitivity of Xpert is lower in children compared to adults and even substantially lower among ambulant paediatric populations compared to paediatric inpatients. (11-17). As such, diagnosis of childhood TB is challenging with the current routine clinical and laboratory diagnostic tools. Thus, there is the widely acknowledged need for a new, preferably point-of-care, diagnostic tool that could result in rapid and accurate diagnosis of TB disease in children.

Research into host TB biomarkers has recently gained more prominence due to the lack of suitable tests based on detection of the pathogen (18), and the potential they offer for translation into a non-sputum based point-of-care (POC) test that could provide fast and accurate classification of TB, which is a critical need in the field of TB (19). Research studies investigating TB biomarkers involve immunological approaches including the use of antigen stimulated peripheral blood, as well as the relatively more advanced ‘*omic*s’ approaches including *transcriptomics, metabolomics, lipidomics* and *proteomic* markers. However, while majority of studies that investigated novel TB biomarkers were carried out in adult populations, research studies investigating TB biomarker discovery in children are currently emerging. It will not be appropriate to extrapolate adult findings to paediatric populations given the considerable heterogeneity in immune responses to *M.tb* between adults and children. For example, the level of cytokines in Quantiferon (QFT) supernatant have been reported to be different even among children of different age groups in the same cohort (20).

**AIMS**: The aim of this systematic review is to evaluate the profile of emerging biomarkers for diagnosis of TB in children, and to compare their diagnostic accuracy to WHO-endorsed target product profile (TPP) for new TB diagnostic tests in children (19).

**Review question (in PICOT format)**:

*Focused research question*: How do diagnostic accuracy of new/emerging biomarkers for childhood TB compare to published TPP for new potential TB diagnostics in children?

**Population**: Children aged less than 15 years

**Intervention**: Host-response and pathogen-derived biomarkers: cytokine signatures in serum or plasma or unstimulated and *M.tb*-antigen stimulated supernatants; cell surface markers; blood transcript signatures; metabolic signatures; proteomic signatures; pathogen-derive cell wall antigens, etc.

**Comparator**: WHO-endorsed target product profile for new TB diagnostic tests in children (19).

**Outcome**: TB diagnosis

**Criteria for considering studies for the review:**

*Types of studies (designs)*: Case-control studies, cross-sectional studies and cohort studies investigating biomarkers that could distinguish pulmonary TB from other respiratory diseases or healthy controls with latent TB infection or healthy uninfected controls.

*Types of participants*: Exclusively paediatric study subjects, defined as age < 15 years

*Types of interventions (or exposures)*: Host-response and pathogen-derived biomarkers: cytokine signatures in serum or plasma or unstimulated and *M.tb*-antigen stimulated supernatants; cell surface markers; blood transcript signatures; metabolic signatures; proteomic signatures; pathogen-derive cell wall antigens, etc.

*Types of outcome measures*:

Primary: Diagnosis of pulmonary TB in children

Secondary:

1. Compare the diagnostic accuracy of biomarkers for childhood TB to published target product profiles recommended for potential new diagnostics for TB in children;
2. Highlight promising biomarkers based on assessment of study quality and diagnostic performance.

**SEARCH METHODS**

*Databases and other sources, time periods, search terms, language restrictions, etc:*

Systematic review of non-DNA biomarkers and multi-marker biosignatures for diagnosis of active tuberculosis in exclusively paediatric study subjects, defined as age less than 15 years, in studies published between January 1, 2000 and November 27, 2017. PubMed, EMBASE, and Web of Science will be searched for relevant publications. The PubMed search term to be used is:

((((tuberculosis[ti] OR TB[ti]) AND (child*[tw] OR pediat*[tw])) ( (("biological markers"[mesh] OR biological marker*[tw] OR biomarker*[tw] OR biosignature*[tw]) NOT (tumour*[tw] OR tumor*[tw] OR "tumor markers, biological"[mesh])) OR (miRNA[tw] OR microRNA[tw] OR proteom*[tw] OR transcriptom*[tw] OR immunoassay*[tw] OR immunoassay[mesh] OR LAM[tw] OR lipoarabinomannan*[tw] OR ("immunologic tests"[mesh] AND diagnos*[tw]) OR ((mycolic acid[tw] OR glycolipid*[tw]) AND (diagnos*[tw] OR detect*[tw])) OR (cytokine*[tw] AND diagnos*[tw]) )) NOT (animals[mesh] NOT humans[mesh])))

In the case of PubMed, searches including both medical subject headings (MeSH) and “text words” will be used. For EMBASE and Web of Science, ‘English’ and ‘Human’ filters will be used. For each database, the search term would be transposed as appropriate.

**REVIEW METHODS**

*Study selection methods:*

Preparation and reporting of our systematic review will be according to the Preferred Reporting Items for Systematic Reviews and Meta-Analysis (PRISMA) guidelines (21). Biomarkers and multi-marker biosignatures, of either host or mycobacterial origin, will be included. Studies using adult or mixed adult and paediatric populations and studies reporting biomarkers for extra-pulmonary TB (EPTB) detection will be excluded. Index tests that required imaging techniques or detection from bacterial culture will be excluded. Studies published in English and French are eligible for inclusion.

*Data extraction methods (including methods for resolving disagreements):*

The form utilized for data extraction was piloted for a separate systematic review (MacLean et al., unpublished) and will be further refined for this systematic review. Publications will be screened by title and abstract by two reviewers (TT and EM) before full-text screening. TT and EM will confer to determine appropriateness of all selected articles. TT and EM will carry out double data extraction using a structured Google form.

*Data items that will be collected:*

1. biomarker: name, biomarker name, number of markers, category of biomarker, up or down regulation of biomarker;
2. index test: sample type needed, type of test, commercialization status, level of technical facilities required, blinding of test;
3. reference standard: reference standard employed and remarks;
4. participant information: descriptive study population information, age demographic, total number of participants, negative population, different populations included in study;
5. study information: author-defined study design, sampling strategy, study location, study time period, place of sample testing, study location, study time period;
6. deployment information: place of sample testing, current level of evidence;
7. diagnostic performance data: numbers of true positives, true negatives, false positives, and false negatives, sensitivity and confidence intervals, specificity and confidence intervals, statistical significance, AUC, cut-off and explanation, positive and negative predictive values, number of TB cases assayed, number of reference standard negative controls
8. bibliographic information

*Quality assessment methods [risk of bias in individual studies] and how quality data will be used:* Study quality will be assessed using specific sets of criteria within four domains of the Quality Assessment of Diagnostic Accuracy Studies – 2 (QUADAS-2) framework (22). As per QUADAS-2 guidelines, the selected questions that are deemed most relevant will be used for identifying biases for studies that included in the review. Each criterion will be classified as either “Yes”, “No”, or “Unclear” when applied to the information that is available in included publications.

*Main summary measures (e.g., risk ratio, difference in means):* **Not applicable; review will not include meta-analysis**.

*Data synthesis and meta-analysis methods*: **Not applicable**

*Heterogeneity assessment:* **Not Applicable**

*Additional analyses (e.g. sensitivity or subgroup analyses, meta-regression)*: **Not Applicable**

*Assessment of publication bias (funnel plot, Egger test, etc):* **Not Applicable**

**References:**

1. Marais BJ, Hesseling AC, Gie RP, Schaaf HS, Beyers N. The burden of childhood tuberculosis and the accuracy of community-based surveillance data. Int J Tuberc Lung Dis. 2006;10(3):259-63.

2. Nelson LJ, Wells CD. Global epidemiology of childhood tuberculosis. Int J Tuberc Lung Dis. 2004;8(5):636-47.

3. Newton SM, Brent AJ, Anderson S, Whittaker E, Kampmann B. Paediatric tuberculosis. The Lancet infectious diseases. 2008;8(8):498-510.

4. Tahmeed Ahmed FS, A.M. Shamsir Ahmed. Childhood Tuberculosis: A review of Epidemiology, Diagnosis and Management. Inf Dis J of Pakistan. 2008;17:52-60.

5. Nicol MP, Zar HJ. New specimens and laboratory diagnostics for childhood pulmonary TB: progress and prospects. Paediatr Respir Rev. 2011;12(1):16-21.

6. Edwards DJ, Kitetele F, Van Rie A. Agreement between clinical scoring systems used for the diagnosis of pediatric tuberculosis in the HIV era. Int J Tuberc Lung Dis. 2007;11(3):263-9.

7. Nelson LJ, Schneider E, Wells CD, Moore M. Epidemiology of childhood tuberculosis in the United States, 1993-2001: the need for continued vigilance. Pediatrics. 2004;114(2):333-41.

8. Nicol MP, Pienaar D, Wood K, Eley B, Wilkinson RJ, Henderson H, et al. Enzyme-linked immunospot assay responses to early secretory antigenic target 6, culture filtrate protein 10, and purified protein derivative among children with tuberculosis: implications for diagnosis and monitoring of therapy. Clinical infectious diseases : an official publication of the Infectious Diseases Society of America. 2005;40(9):1301-8.

9. Boehme CC, Nicol MP, Nabeta P, Michael JS, Gotuzzo E, Tahirli R, et al. Feasibility, diagnostic accuracy, and effectiveness of decentralised use of the Xpert MTB/RIF test for diagnosis of tuberculosis and multidrug resistance: a multicentre implementation study. Lancet. 2011;377(9776):1495-505.

10. Theron G, Peter J, van Zyl-Smit R, Mishra H, Streicher E, Murray S, et al. Evaluation of the Xpert MTB/RIF assay for the diagnosis of pulmonary tuberculosis in a high HIV prevalence setting. American journal of respiratory and critical care medicine. 2011;184(1):132-40.

11. Nicol MP, Workman L, Isaacs W, Munro J, Black F, Eley B, et al. Accuracy of the Xpert MTB/RIF test for the diagnosis of pulmonary tuberculosis in children admitted to hospital in Cape Town, South Africa: a descriptive study. The Lancet infectious diseases. 2011;11(11):819-24.

12. Zar HJ, Workman L, Isaacs W, Munro J, Black F, Eley B, et al. Rapid molecular diagnosis of pulmonary tuberculosis in children using nasopharyngeal specimens. Clinical infectious diseases : an official publication of the Infectious Diseases Society of America. 2012;55(8):1088-95.

13. Rachow A, Clowes P, Saathoff E, Mtafya B, Michael E, Ntinginya EN, et al. Increased and expedited case detection by Xpert MTB/RIF assay in childhood tuberculosis: a prospective cohort study. Clinical infectious diseases : an official publication of the Infectious Diseases Society of America. 2012;54(10):1388-96.

14. Sekadde MP, Wobudeya E, Joloba ML, Ssengooba W, Kisembo H, Bakeera-Kitaka S, et al. Evaluation of the Xpert MTB/RIF test for the diagnosis of childhood pulmonary tuberculosis in Uganda: a cross-sectional diagnostic study. BMC infectious diseases. 2013;13:133.

15. Zar HJ, Workman L, Isaacs W, Dheda K, Zemanay W, Nicol MP. Rapid diagnosis of pulmonary tuberculosis in African children in a primary care setting by use of Xpert MTB/RIF on respiratory specimens: a prospective study. The Lancet Global health. 2013;1(2):e97-104.

16. Detjen AK, DiNardo AR, Leyden J, Steingart KR, Menzies D, Schiller I, et al. Xpert MTB/RIF assay for the diagnosis of pulmonary tuberculosis in children: a systematic review and meta-analysis. The Lancet Respiratory medicine. 2015.

17. Togun TO, Egere U, Sillah AK, Ayorinde A, Mendy F, Tientcheu L, et al. Contribution of Xpert MTB/Rif to the diagnosis of Pulmonary Tuberculosis among TB-exposed Children in The Gambia. Int J Tuberc Lung Dis (Accepted). 2015.

18. Walzl G, Ronacher K, Hanekom W, Scriba TJ, Zumla A. Immunological biomarkers of tuberculosis. Nature reviews Immunology. 2011;11(5):343-54.

19. Denkinger CM, Kik SV, Cirillo DM, Casenghi M, Shinnick T, Weyer K, et al. Defining the Needs for Next Generation Assays for Tuberculosis. The Journal of infectious diseases. 2015;211(suppl 2):S29-S38.

20. Lighter-Fisher J, Peng CH, Tse DB. Cytokine responses to QuantiFERON(R) peptides, purified protein derivative and recombinant ESAT-6 in children with tuberculosis. Int J Tuberc Lung Dis. 2010;14(12):1548-55.

21. Moher D, Liberati A, Tetzlaff J, Altman DG, Group P. Preferred reporting items for systematic reviews and meta-analyses: the PRISMA statement. Journal of clinical epidemiology. 2009;62(10):1006-12.

22. Whiting PF, Rutjes AW, Westwood ME, Mallett S, Deeks JJ, Reitsma JB, et al. QUADAS-2: a revised tool for the quality assessment of diagnostic accuracy studies. Annals of internal medicine. 2011;155(8):529-36.
