# Supplementary material for: Biomarkers for diagnosis of childhood tuberculosis: A systematic review
Source: PLoS One. 2018 Sep 13;13(9):e0204029. doi: 10.1371/journal.pone.0204029 (PMC6136789; doi:10.1371/journal.pone.0204029)
Supplement: S2 File — List of the fields for data extraction and structured google form used for data extraction. (PDF) [file pone.0204029.s002.pdf]

Biomarkers for diagnosis of childhood tuberculosis: a systematic review

Toyin Togun[1]*, Emily MacLean[1], Beate Kampmann[2,3], and Madhukar Pai[1,4]

Supplementary Information:

Information extracted from each study comprised the following:

(i) biomarker: name, biomarker name, number of markers, category of biomarker, up or down regulation of biomarker;

(ii) index test: sample type needed, type of test, commercialization status, level of technical facilities required, blinding of test;

(iii) reference standard: reference standard employed and remarks;

(iv) participant information: descriptive study population information, age demographic, total number of participants, negative population, different populations included in study;

(v) study information: author-defined study design, sampling strategy, study location, study time period, place of sample testing, study location, study time period;

(vi) deployment information: place of sample testing, current level of evidence;

(vii) diagnostic performance data: numbers of true positives, true negatives, false positives, and false negatives, sensitivity and confidence intervals, specificity and confidence intervals, statistical significance, AUC, cut-off and explanation, positive and negative predictive values, number of TB cases assayed, number of reference standard negative controls

(viii) bibliographic information

Biomarker & primary study information

biomarker information and bibliographic info from primary studies

* Required

BIOMARKER & STUDY INFORMATION

1. **Biomarker name? (001)** *

2. **Who completed this form?**
Mark only one oval.
   - Emily (1)
   - Toyin (2)
   - Other:

3. **Alternate biomarker name (002)**
other names the biomarker is called, separate with ;

4. **Number of markers (003)** *

5. **Title of publication (004)** *

6. **Year of publication (005)** *

7. **First author (006)** *
i.e. First name Last name (e.g. Jacqueline M. Achkar)

8. **Corresponding author name (007)**
i.e. First name Last name (e.g. Jacqueline M. Achkar)

9. **Corresponding author country (008)**

10. **Corresponding author email (009)**

11. **Study design (016)**
Cohort (group of individuals, initially defined and composed, with common characteristics (e.g., condition, birth year), who are examined or traced over a given time period) / Case-control (group of individuals with specific characteristics (e.g., conditions or exposures) compared to group(s) with different characteristics, but otherwise similar) usually not representative of the whole population / Cross-sectional: a cross-sectional study (also known as a cross-sectional analysis, transversal study, prevalence study) is a type of observational study that involves the analysis of data collected from a population, or a representative subset, at one specific point in time from: https://clinicaltrials.gov/ct2/home; Unclear requireds discussion; NR=Not reported
Mark only one oval.

- Cohort (1)
- Case control (2)
- Cross-sectional (3)
- NR (4)
- Unclear! (5)
- Other: ____________

12. **Sampling (017)**
Consecutive Sample=Sequential Sample: Sample in which the units are chosen on a strict first come first chosen basis. All subjects who are eligible should be included as they are seen. / Convenience Sample: Subjects or groups selected at the investigator's convenience or primarily because they were available at a convenient time or place. / Random Sample: A sample derived by selecting sampling units (eg, individualpatients) such that each unit has an independent and fixed (generally equal) chance ofselection. Whether a given unit is selected is determined by chance (eg, by a tablerandomly ordered numbers).
Mark only one oval.

- Convenience (1)
- Consecutive (2)
- Random (3)
- NR (4)
- Unclear! (5)
- Other: ____________

13. **Study period (018)**
month/year TO month/year; e.g. 3/2011-5/2013
(use 0/2011 if month unclear, no blanks)

14. **Sample/patient location (019)**
Country only, separate with ;

15. **Type of sample for index test? (020)**
Check all that apply.

- [ ] Breath (2)
- [ ] PBMCs
- [ ] Plasma (9)
- [ ] QFT supernatant
- [ ] Saliva (4)
- [ ] Serum (8)
- [ ] Sputum (6)
- [ ] Urine (7)
- [ ] WBA Supernatant (10)
- [ ] Whole blood (1)
- [ ] Other: ____________________

16. **Type of biomarker (021)**
Check all that apply.

- [ ] Antibody (1)
- [ ] Cell surface protein (2)
- [ ] Cytokine (3)
- [ ] Enzyme (4)
- [ ] Hematological marker (5)
- [ ] Metabolic activity marker (6)
- [ ] Lipid (7)
- [ ] microRNA (8)
- [ ] mRNA expression/host transcription (9)
- [ ] TB protein: antigenic (10)
- [ ] TB protein: enzyme (11)
- [ ] TB: lipid (12)
- [ ] TB: mycolic acid (13)
- [ ] TB: transcriptional marker (14)
- [ ] VOC (15)
- [ ] Whole bacili (16)
- [ ] Unclear! (17)
- [ ] Gene transcript
- [ ] Other: ____________________

17. **Index test type (method of detection) (022)**
Check all that apply.

- [ ] Cell stimulation (1)
- [ ] Cytometry (FACS or otherwise) (2)
- [ ] Electronic nose (3)
- [ ] MS (6)
- [ ] Microarray (7)
- [ ] NMR (8)
- [ ] PCR (9)
- [ ] Multiplex
- [ ] ELISA
- [ ] Lateral flow assay
- [ ] Other immunoassays
- [ ] Other: ____________________

18. **Index method details (023)**
e.g. type of cells a cell marker is on; type of Ag used for cell stimulation; model used; etc

____
____
____
____
____

19. **Was the index test interpreted in a blinded manner? (025)**
Mark only one oval.

- ( ) Yes (1)
- ( ) No (2)
- ( ) NR (3)
- ( ) Other: ____________________

20. **Study Population Section (027)**
Copy author defined study population section; include disease groups and controls

____
____
____
____
____

21. **Patient subgroup analysis [patient phenotype] (072)**

____

22. **Total no. enrolled patients (029)**
after exclusion, (Largest number minus exclusions)

____

23. **Reference standard used (030)**
to calculate diagnostic performance (e.g Positive, Negative), multiple means composite
Check all that apply.

- [ ] Liquid culture (1)
- [ ] Solid culture (2)
- [ ] Culture (non specified) (3)
- [ ] Smear microscopy (4)
- [ ] Chest x-ray (5)
- [ ] Xpert (6)
- [ ] Molecular (not including Xpert, e.g. sequencing, LPA, qPCR, etc) (7)
- [ ] Clinical signs (8)
- [ ] Outcome (9)
- [ ] TB treatment regimen initiated (10)
- [ ] Cytometry (flow or other) (11)
- [ ] NR (12)
- [ ] Other: ______________________________

24. **Remarks on Reference standard (031)**
Sub-group analysis, different reference standards?

____
____
____
____
____

25. **Patient characteristics (032)**
(Check if yes)
Check all that apply.

- [ ] HIV co-infection (1)
- [ ] BCG vaccination (2)
- [ ] Latent TB infection (3)
- [ ] Extrapulmonary TB infection (4)
- [ ] Smear status (5)
- [ ] History of prior TB (6)
- [ ] Other immuno-compromised condition (7)
- [ ] Chemo prophylaxis (8)
- [ ] None (9)
- [ ] Other: ____________________

26. **Negative Population (033)**
Status of individuals used for comparison (reference test negative patients). Was the enrolment based on TB suspects (thus negatives have other respiratory disases >> ORD) or individuals with other diseases/sympthoms or did the study use healthy controls (endemic vs. non-endemic)? Select multiple if combinations were used to calculate the diagnostic performance (as reported below).
Check all that apply.

- [ ] (OD) Other disease (1)
- [ ] (ORD) Other respiratory disease (2)
- [ ] Healthy endemics (3)
- [ ] Healthy non-endemics (4)
- [ ] Disease contacts (5)
- [ ] LTBI (6)
- [ ] Other: ____________________

DIAGNOSTIC ACCURACY: PERFORMANCE DATA

27. **TP (True Positives) (035)**

____

28. **FN (False Negatives) (036)**
Sick-TP

____

29. **TN (True Negatives) (037)**

____

30. **FP (False Positives) (038)**
Healthy-TN

____

31. **Sensitivity (not percentage) (039)**
sensitivity, example 0.956, use NR for not reported

32. **Confidence interval of sensitivity (040)**
i.e. [x,x], example [0.942,0.956]

33. **Specificity (not percentage) (041)**
specificity, e.g. 0.843, use NR for not reported

34. **Confidence interval of specificity (042)**
i.e. [x,x]

35. **Statistical significance (p-value) (043)**
full p-value statement, e.g. p<0.01; p=0.05, use NR for not reported

36. **Area Under ROC Curve (044)**
example 0.867, use NR for not reported

37. **Was the chosen point on the ROC curve pre-defined? (071)**
Mark only one oval.

- Yes (1)
- No (2)

38. **Explanatory notes re: justification of curve point (064)**

39. **Sick (#Positive reference standard) (045)**
Total number of sick patients based on reference standard (=Positive Reference Standard Results), Value only needed if study did not report on TP, FP, TN, FP

40. **Healthy (#Negative reference standard) (050)**

Total number of patients without TB based on reference standard (=Negative Reference Standards Results), Value only needed if study did not report on TP, FP, TN, FP

41. **PPV (051)**

= TP / (TP + FP), use NR for not reported, Value only needed if study did not report on TP, FP, TN, FP

42. **NPV (052)**

= TN / (TN + FN), use NR for not reported, Value only needed if study did not report on TP, FP, TN, FP

43. **Expression of biomarkers**

= either upregulated or downregulated in TB cases or controls

Powered by
Google Forms
